# Supplementary material for: HIVprotI: an integrated web based platform for prediction and design of HIV proteins inhibitors
Source: J Cheminform. 2018 Mar 9;10:12. doi: 10.1186/s13321-018-0266-y (PMC5845081; doi:10.1186/s13321-018-0266-y)
Supplement: Supplementary file 2 — Additional file 2. Source code of HIVProtI web server. [file 13321_2018_266_MOESM2_ESM.zip › HIVprotI_Source-code/hivprotI/weka-3-6-6/doc/weka/attributeSelection/LatentSemanticAnalysis.html]

LatentSemanticAnalysis


---


|  |  |  |
| --- | --- | --- |
| **Overview** \| **Package** \| **Class** \| **Tree** \| **Deprecated** \| **Index** \| **Help** \| **Weka's home** | |  |
| **PREV CLASS**   **NEXT CLASS** | **FRAMES**    **NO FRAMES**     **All Classes** |  |
| SUMMARY: NESTED \| FIELD \| CONSTR \| METHOD | DETAIL: FIELD \| CONSTR \| METHOD |  |


---


## weka.attributeSelection Class LatentSemanticAnalysis

```
java.lang.Object
  weka.attributeSelection.ASEvaluation
      weka.attributeSelection.UnsupervisedAttributeEvaluator
          weka.attributeSelection.LatentSemanticAnalysis
```

**All Implemented Interfaces:**: java.io.Serializable, AttributeEvaluator, AttributeTransformer, CapabilitiesHandler, OptionHandler, RevisionHandler

---

``` public class LatentSemanticAnalysis extends UnsupervisedAttributeEvaluator implements AttributeTransformer, OptionHandler ```

Performs latent semantic analysis and transformation of the data.
Use in conjunction with a Ranker search. A low-rank approximation
of the full data is found by specifying the number of singular values
to use. The dataset may be transformed to give the relation of either
the attributes or the instances (default) to the concept space created
by the transformation.


Valid options are:

```
 -N
  Normalize input data.
```

```
 -R
  Rank approximation used in LSA. May be actual number of 
  LSA attributes to include (if greater than 1) or a proportion 
  of total singular values to account for (if between 0 and 1). 
  A value less than or equal to zero means use all latent variables.
  (default = 0.95)
```

```
 -A
  Maximum number of attributes to include in 
  transformed attribute names. (-1 = include all)
```

**Version:**
:   $Revision: 5728 $

**Author:**
:   Amri Napolitano

**See Also:**: Serialized Form

---

**Constructor Summary**

`LatentSemanticAnalysis()`


| **Method Summary** | |
| --- | --- |
| `void` | `buildEvaluator(Instances data)`             Initializes the singular values/vectors and performs the analysis |
| `Instance` | `convertInstance(Instance instance)`             Transform an instance in original (unnormalized) format |
| `double` | `evaluateAttribute(int att)`             Evaluates the merit of a transformed attribute. |
| `Capabilities` | `getCapabilities()`             Returns the capabilities of this evaluator. |
| `int` | `getMaximumAttributeNames()`             Gets maximum number of attributes to include in transformed attribute names. |
| `boolean` | `getNormalize()`             Gets whether or not input data is to be normalized |
| `java.lang.String[]` | `getOptions()`             Gets the current settings of LatentSemanticAnalysis |
| `double` | `getRank()`             Gets the desired matrix rank (or coverage proportion) for feature-space reduction |
| `java.lang.String` | `getRevision()`             Returns the revision string. |
| `java.lang.String` | `globalInfo()`             Returns a string describing this attribute transformer |
| `java.util.Enumeration` | `listOptions()`             Returns an enumeration describing the available options. |
| `static void` | `main(java.lang.String[] argv)`             Main method for testing this class |
| `java.lang.String` | `maximumAttributeNamesTipText()`             Returns the tip text for this property |
| `java.lang.String` | `normalizeTipText()`             Returns the tip text for this property |
| `java.lang.String` | `rankTipText()`             Returns the tip text for this property |
| `void` | `setMaximumAttributeNames(int newMaxAttributes)`             Sets maximum number of attributes to include in transformed attribute names. |
| `void` | `setNormalize(boolean newNormalize)`             Set whether input data will be normalized. |
| `void` | `setOptions(java.lang.String[] options)`             Parses a given list of options. |
| `void` | `setRank(double newRank)`             Sets the desired matrix rank (or coverage proportion) for feature-space reduction |
| `java.lang.String` | `toString()`             Returns a description of this attribute transformer |
| `Instances` | `transformedData(Instances data)`             Transform the supplied data set (assumed to be the same format as the training data) |
| `Instances` | `transformedHeader()`             Returns just the header for the transformed data (ie. |

**Methods inherited from class weka.attributeSelection.ASEvaluation**

`forName, makeCopies, postProcess`

**Methods inherited from class java.lang.Object**

`equals, getClass, hashCode, notify, notifyAll, wait, wait, wait`

**Constructor Detail**

### LatentSemanticAnalysis

```
public LatentSemanticAnalysis()
```


**Method Detail**

### globalInfo

```
public java.lang.String globalInfo()
```

:   Returns a string describing this attribute transformer

    :   **Returns:**: a description of the evaluator suitable for displaying in the explorer/experimenter gui

---


### listOptions

```
public java.util.Enumeration listOptions()
```

:   Returns an enumeration describing the available options.

    :   **Specified by:**: `listOptions` in interface `OptionHandler`
    :   **Returns:**: an enumeration of all the available options.

---


### setOptions

```
public void setOptions(java.lang.String[] options)
                throws java.lang.Exception
```

:   Parses a given list of options.


    Valid options are:

    ```
     -N
      Normalize input data.
    ```

    ```
     -R
      Rank approximation used in LSA. May be actual number of 
      LSA attributes to include (if greater than 1) or a proportion 
      of total singular values to account for (if between 0 and 1). 
      A value less than or equal to zero means use all latent variables.
      (default = 0.95)
    ```

    ```
     -A
      Maximum number of attributes to include in 
      transformed attribute names. (-1 = include all)
    ```

    :   **Specified by:**: `setOptions` in interface `OptionHandler`
    :   **Parameters:**: `options` - the list of options as an array of strings **Throws:**: `java.lang.Exception` - if an option is not supported

---


### normalizeTipText

```
public java.lang.String normalizeTipText()
```

:   Returns the tip text for this property

    :   **Returns:**: tip text for this property suitable for displaying in the explorer/experimenter gui

---


### setNormalize

```
public void setNormalize(boolean newNormalize)
```

:   Set whether input data will be normalized.

    :   **Parameters:**: `newNormalize` - true if input data is to be normalized

---


### getNormalize

```
public boolean getNormalize()
```

:   Gets whether or not input data is to be normalized

    :   **Returns:**: true if input data is to be normalized

---


### rankTipText

```
public java.lang.String rankTipText()
```

:   Returns the tip text for this property

    :   **Returns:**: tip text for this property suitable for displaying in the explorer/experimenter gui

---


### setRank

```
public void setRank(double newRank)
```

:   Sets the desired matrix rank (or coverage proportion) for feature-space reduction

    :   **Parameters:**: `newRank` - the desired rank (or coverage) for feature-space reduction

---


### getRank

```
public double getRank()
```

:   Gets the desired matrix rank (or coverage proportion) for feature-space reduction

    :   **Returns:**: the rank (or coverage) for feature-space reduction

---


### maximumAttributeNamesTipText

```
public java.lang.String maximumAttributeNamesTipText()
```

:   Returns the tip text for this property

    :   **Returns:**: tip text for this property suitable for displaying in the explorer/experimenter gui

---


### setMaximumAttributeNames

```
public void setMaximumAttributeNames(int newMaxAttributes)
```

:   Sets maximum number of attributes to include in
    transformed attribute names.

    :   **Parameters:**: `newMaxAttributes` - the maximum number of attributes

---


### getMaximumAttributeNames

```
public int getMaximumAttributeNames()
```

:   Gets maximum number of attributes to include in
    transformed attribute names.

    :   **Returns:**: the maximum number of attributes

---


### getOptions

```
public java.lang.String[] getOptions()
```

:   Gets the current settings of LatentSemanticAnalysis

    :   **Specified by:**: `getOptions` in interface `OptionHandler`
    :   **Returns:**: an array of strings suitable for passing to setOptions()

---


### getCapabilities

```
public Capabilities getCapabilities()
```

:   Returns the capabilities of this evaluator.

    :   **Specified by:**: `getCapabilities` in interface `CapabilitiesHandler` **Overrides:**: `getCapabilities` in class `ASEvaluation`
    :   **Returns:**: the capabilities of this evaluator **See Also:**: `Capabilities`

---


### buildEvaluator

```
public void buildEvaluator(Instances data)
                    throws java.lang.Exception
```

:   Initializes the singular values/vectors and performs the analysis

    :   **Specified by:**: `buildEvaluator` in class `ASEvaluation`
    :   **Parameters:**: `data` - the instances to analyse/transform **Throws:**: `java.lang.Exception` - if analysis fails

---


### transformedHeader

```
public Instances transformedHeader()
                            throws java.lang.Exception
```

:   Returns just the header for the transformed data (ie. an empty
    set of instances. This is so that AttributeSelection can
    determine the structure of the transformed data without actually
    having to get all the transformed data through getTransformedData().

    :   **Specified by:**: `transformedHeader` in interface `AttributeTransformer`
    :   **Returns:**: the header of the transformed data. **Throws:**: `java.lang.Exception` - if the header of the transformed data can't be determined.

---


### transformedData

```
public Instances transformedData(Instances data)
                          throws java.lang.Exception
```

:   Transform the supplied data set (assumed to be the same format
    as the training data)

    :   **Specified by:**: `transformedData` in interface `AttributeTransformer`
    :   **Returns:**: the transformed training data **Throws:**: `java.lang.Exception` - if transformed data can't be returned

---


### evaluateAttribute

```
public double evaluateAttribute(int att)
                         throws java.lang.Exception
```

:   Evaluates the merit of a transformed attribute. This is defined
    to be the square of the singular value for the latent variable
    corresponding to the transformed attribute.

    :   **Specified by:**: `evaluateAttribute` in interface `AttributeEvaluator`
    :   **Parameters:**: `att` - the attribute to be evaluated **Returns:**: the merit of a transformed attribute **Throws:**: `java.lang.Exception` - if attribute can't be evaluated

---


### convertInstance

```
public Instance convertInstance(Instance instance)
                         throws java.lang.Exception
```

:   Transform an instance in original (unnormalized) format

    :   **Specified by:**: `convertInstance` in interface `AttributeTransformer`
    :   **Parameters:**: `instance` - an instance in the original (unnormalized) format **Returns:**: a transformed instance **Throws:**: `java.lang.Exception` - if instance can't be transformed

---


### toString

```
public java.lang.String toString()
```

:   Returns a description of this attribute transformer

    :   **Overrides:**: `toString` in class `java.lang.Object`
    :   **Returns:**: a String describing this attribute transformer

---


### getRevision

```
public java.lang.String getRevision()
```

:   Returns the revision string.

    :   **Specified by:**: `getRevision` in interface `RevisionHandler` **Overrides:**: `getRevision` in class `ASEvaluation`
    :   **Returns:**: the revision

---


### main

```
public static void main(java.lang.String[] argv)
```

:   Main method for testing this class

    :   **Parameters:**: `argv` - should contain the command line arguments to the evaluator/transformer (see AttributeSelection)


---


|  |  |  |
| --- | --- | --- |
| **Overview** \| **Package** \| **Class** \| **Tree** \| **Deprecated** \| **Index** \| **Help** \| **Weka's home** | |  |
| **PREV CLASS**   **NEXT CLASS** | **FRAMES**    **NO FRAMES**     **All Classes** |  |
| SUMMARY: NESTED \| FIELD \| CONSTR \| METHOD | DETAIL: FIELD \| CONSTR \| METHOD |  |


---
